# Cochlear Lymph Fluid Signal Increase in Patients with Otosclerosis after Intravenous Administration of Gadodiamide

**DOI:** 10.2463/mrms.mp.2015-0121

**Published:** 2016-02-03

**Authors:** Shinji NAGANAWA, Hisashi KAWAI, Toshiaki TAOKA, Kojiro SUZUKI, Shingo IWANO, Hiroko SATAKE, Michihiko SONE, Mitsuru IKEDA

**Affiliations:** 1 Department of Radiology, Nagoya University Graduate School of Medicine 65 Tsurumai-cho, Shouwa-ku, Nagoya, Aichi 466-8550, Japan; 2 Department of Otorhinolaryngology, Nagoya University Graduate School of Medicine; 3 Department of Radiological and Medical Laboratory Sciences, Nagoya University Graduate School of Medicine

**Keywords:** magnetic resonance imaging, otosclerosis, temporal bone disease

## Abstract

**Purpose:**

Increased cochlear lymph fluid signals on three-dimensional fluid-attenuated inversion recovery (3D-FLAIR) images obtained several minutes after intravenous administration of a single dose of gadolinium-based contrast agent (IV-SD-GBCA) in a patient with severe retrofenestral type otosclerosis had been reported. This increase was thought to represent breakdown of the blood-labyrinthine barrier. The purpose of this study was to evaluate cochlear lymph signal on heavily T_2_-weighted 3D-FLAIR (HF) images obtained 4 hours after IV-SD-GBCA in patients with otosclerosis, Ménière’s disease, and healthy subjects.

**Materials and Methods:**

Twenty-two ears from 12 patients with otosclerotic plaques determined by computed tomography (CT), 16 ears from 8 healthy volunteers, and 10 ears from 9 Ménière’s disease patients with significant endolymphatic hydrops on magnetic resonance (MR) images were retrospectively analyzed. Images were obtained 4 hours after IV-SD-GBCA. Patients and healthy volunteers underwent MR cisternography (MRC) for anatomical reference of the fluid space and HF at 3T. The region of interest (ROI) was manually drawn on MRC images around the scala tympani in the basal cochlear turn. The reference ROI was set in the cerebellum. ROIs were copied onto HF images and the signal intensity ratio (SIR) of cochlear perilymph to cerebellum was measured. Differences in the SIR on HF images among the three groups were tested by one-way analysis of variance (ANOVA).

**Results:**

The mean SIR was 24.0 ± 10.1 in otosclerosis patients, 7.9 ± 1.5 in volunteers, and 11.6 ± 3.9 in Ménière’s disease patients. The mean SIR was significantly higher in the otosclerosis group than in the other groups (*P* < 0.001). In the otosclerosis group, there was a significant difference in the SIR between the retrofenestral type and the fenestral type (*P* = 0.033).

**Conclusions:**

In patients with otosclerosis, the SIR was higher than in Ménière’s disease patients or in healthy volunteers. The SIR was higher in the retrofenestral type than in the fenestral type.

## Introduction

Otosclerosis is an otodystrophy of the otic capsule and is a cause of conductive, mixed, or sensorineural hearing loss. Otosclerosis is usually categorized into two types, fenestral and retrofenestral. These two types represent a continuum rather than two separate entities.^1,2^ Imaging plays an important role in the diagnosis and management of otosclerosis. High resolution computed tomography (HRCT) is the modality of choice for assessment of the labyrinthine windows and cochlear capsules.^3^ However, magnetic resonance (MR) imaging has the advantage of being able to assess the labyrinthine lumen.

There was a report of an increased signal in the cochlear lymph fluid on contrast-enhanced three-dimensional fluid-attenuated inversion recovery (3D-FLAIR) images several minutes after the administration of gadolinium-based contrast agents (GBCAs) in a patient with severe retrofenestral otosclerosis (OS), suggesting increased permeability of the blood-labyrinthine barrier in the otosclerosis.^4^

Recently, the applicability of contrast-enhanced T_1_-weighted imaging as a method for monitoring the activity of otospongiotic lesions before and after treatment with sodium alendronate or sodium fluoride has been reported.^5^ However, it is not always possible to identify small otospongiotic lesions on contrast-enhanced T_1_-weighted images in the fenestral condition. Third-generation bisphosphonates are expected to show more powerful treatment effects for otosclerosis.^6^ Thus, in order to aid the development of more effective drug therapies, identifying other reliable and sensitive imaging biomarkers for monitoring the activity of otosclerosis would be of great value.

Endolymphatic hydrops (EHs) has been reported in cases with otosclerosis, and preoperative EH has been suggested as a risk factor for inner ear disturbances after stapes surgery.^7^ EH can be assessed clinically utilizing heavily T_2_-weighted 3D-FLAIR (HF) images obtained 4 hours after intravenous administration of double dose of gadolinium-based contrast agent^8^ or an intravenously administered single dose GBCA (IV-SD-GBCA).^9–18^ At our institution, we routinely evaluated EH in patients with otosclerosis scheduled for stapes surgery by IV-SD-GBCA.

It has been reported that permeability of the blood-labyrinthine barrier is slightly increased in ears diagnosed with Ménière’s disease.^19^ Furthermore, permeability of the blood-labyrinthine barrier has also been reported to increase in other disease conditions, such as sudden deafness,^20,21^ vestibular schwannoma,^22^ labyrinthine invasion of cholesteatoma,^23,24^ and relapsing polychondritis^25^ among others. It is possible that in addition to endolymphatic hydrops, increased permeability of the blood-labyrinthine barrier might also contribute to sensorineural hearing loss in otosclerosis.^7^

HF obtained 4 hours after IV-SD-GBCA is quite sensitive to subtle changes in fluid T_1_-values; in other words, it is quite sensitive to subtle alterations of blood-labyrinthine barrier permeability. Thus, evaluation of blood-labyrinthine barrier permeability may contribute to our basic knowledge and understanding of otosclerosis.

The purpose of this retrospective study was to compare the cochlear lymph fluid signal on HF images obtained 4 hours after IV-SD-GBCA between ears with otosclerosis, Ménière’s disease, and normal ears.

## Materials and Methods

### Patients and volunteers

Between June 1, 2012 and March 30, 2015, 12 consecutive patients (7 men, 5 women; age 35–66 years) with bilateral otosclerosis previously diagnosed on HRCT underwent evaluation for endolymphatic hydrops at 3 Tesla 4 hours after IV-SD-GBCA. Ten ears from five patients had been diagnosed as having retrofenestral otosclerosis based on HRCT and the rest had been diagnosed as having fenestral otosclerosis. Two ears with fenestral otosclerosis had undergone stapes surgery and were excluded; the remaining 22 ears were evaluated. All 22 ears showed mixed hearing loss.

Ten ears from nine patients with Ménière’s disease (5 men, 4 women; age 24–75 years) were included for comparison. These patients were randomly extracted from the radiology database of our department using the following inclusion criteria: (1) patients scanned between June 1, 2012 and March 30, 2015 with clinical suspicion of Ménière’s disease and (2) ears with significant endolymphatic hydrops in both the cochlea and vestibule which were detected by MR imaging.

Sixteen ears from eight healthy male volunteers (age 29–53 years) that had been scanned using the same imaging protocol were included for comparison as controls. A list of ears analyzed in the patient and volunteer groups is shown in Table 1.

For the volunteer scans, the medical ethics committee of our institution approved the study of healthy volunteers and written informed consent was obtained from all volunteers. The retrospective patient study was approved by the medical ethics committee of our institution with a waiver of written informed consent obtained from all patients.

### MR imaging

All MR imaging was performed on a 3-tesla scanner (Verio, Siemens, Erlangen, Germany) using a 32-channel array head coil. MR scanning was performed 4 hours after IV-SD-GBCA (0.2 mL/kg or 0.1 mmol/kg body weight) to evaluate the size of the endolymphatic space in all patients and volunteers. For patients, gadolinium-diethylenetriamine pentaacetic acid-bis (methylamide) (gadodiamide, Gd-DTPA-BMA; Omniscan, Daiichi-Sankyo Co. Ltd., Tokyo, Japan) was utilized. For volunteers, gadoteridol (Gd-HP-DO3A: ProHance, Eisai, Tokyo, Japan) was utilized.

All patients who underwent IV administration had an estimated glomerular filtration rate (eGFR) value exceeding 60 mL/min/1.73 m^2^ .

According to the clinical protocol used by our hospital for the evaluation of endolymphatic hydrops, all patients and volunteers underwent heavily T_2_-weighted MR cisternography (MRC) for anatomical reference of the total lymph fluid and HF with a 2250-msec inversion time (positive perilymph image, PPI) 4 hours after receiving IV-SD-GBCA.^26^

Detailed scan parameters for MRC included: heavily T_2_-weighted MRC images using variable flip angle 3D turbo spin echo technique (SPACE: sampling perfection with application-optimized contrasts using different flip angle evolutions); repetition time (TR), 4400 msec; echo time (TE), 544 msec; frequency-selective fat-suppression pre-pulse; initial re-focusing 180° flip angle rapidly decreased to constant 120° flip angle for the refocusing echo train; echo-train length, 173 with restore magnetization pulse (fast recovery pulse); matrix size, 322 × 384; 96 axial slices of 1.0 mm-thickness covering the labyrinth; field of view (FOV), 15 × 18 cm; generalized autocalibrating partially parallel acquisition (GRAPPA) parallel imaging technique; acceleration factor, 2; number of excitations (NEX), 1.8; and scan time, 2.9 minutes.

The HF (PPI) imaging utilized similar parameters as for the MRC except: TR, 9000 ms; inversion time, 2250 ms, without restore magnetization pulse; 4 excitations, and scan time of 14 minutes.

For the evaluation of endolymphatic hydrops, we generated *“*HYDROPS2 (HYbriD of Reversed image Of MR cisternography and positive Perilymph Signal by heavily T_2_-weighted 3D-FLAIR)” images on the scanner console by subtracting the MRC multiplied by 0.04 from the PPI.^15^ For the purpose of subtraction, negative signal values were allowed. Acquisition of source images took approximately 17 minutes for HYDROPS2 images. During subtraction, we applied no image registration program.

### Image analysis

Images were evaluated quantitatively by a neuroradiologist with 26 years of experience in the field of clinical MRI using a PACS viewer (Rapid-eye, Toshiba, Tokyo, Japan). Morphological distortion of the scala tympani due to enlargement of the scala media (endolymphatic hydrops) is less than that of the scala vestibuli. Contouring of the scala tympani in the basal turn of the cochlea is easy and stable regardless of the degree of endolymphatic hydrops. Therefore, measurements of the lymph fluid signal were performed for the scala tympani in the basal turn of the cochlea.^12^

**Fig. 1.**
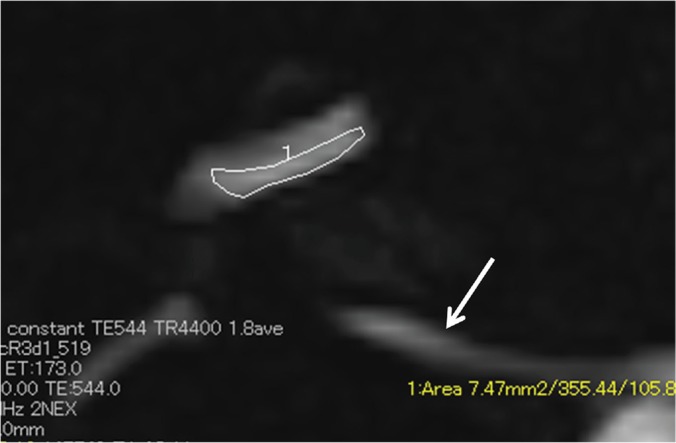
An example of the region of interest (ROI) drawn around the scala tympani of the basal turn of the cochlea on magnetic resonance cisternography (MRC). Arrow indicates the cochlear aqueduct. This image was obtained with sampling perfection with application-optimized contrasts using different flip angle evolution (SPACE) sequence in a patient with Ménière’s disease.

For the scala tympani region of interest (ROI), the slices in which the cochlear aqueduct was visually longest were selected (Fig. 1).

For quantitative evaluation, the signal intensity ratio (SIR) between the signal of the scala tympani and cerebellum was measured on HF images. The ROI for the cerebellum was set as a circle with a diameter of 10 mm in the ipsilateral cerebellar white matter on the MRC images. The ROI for the scala tympani was drawn manually on the MRC images. ROIs were copied and pasted onto HF images.

### Statistical analysis

**Fig. 2.**
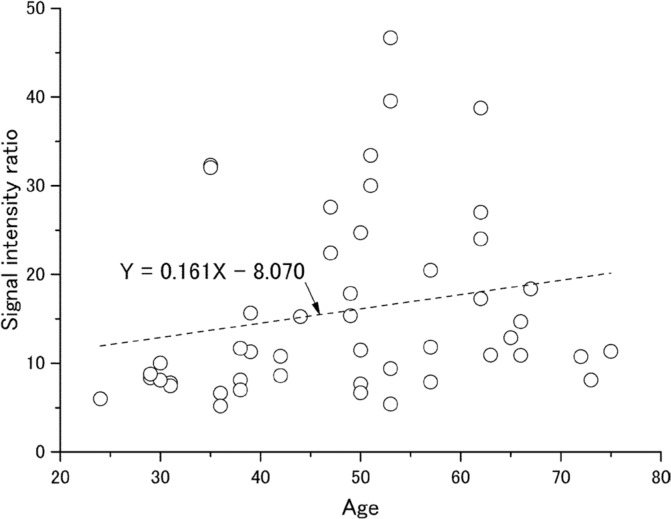
Scattergram between age and the signal intensity ratio (SIR). No significant correlation was found between age and SIR (r^2^ = 0.043).

We first confirmed that there was no linear relationship between age and SIR: Pearson’s correlation coefficient (*r*) was 0.207 (*P* = 0.158) (Fig. 2).

Differences in the SIR among the volunteer, otosclerosis, and Ménière’s disease groups were tested by one-way analysis of variance (ANOVA) and Scheffé post hoc analysis or, when the null hypothesis of equal variance was rejected, by Tamhane post hoc analysis. Further, the SIR was also compared between the fenestral and retrofenestral types in the otosclerosis group using the Student’s *t* test.

We used SPSS 22 software (SPSS Inc., Chicago, IL, USA) for all statistical analyses, and adopted 5% as the significance level for the statistical test.

## Results

The results of SIR measurement are shown in Tables 1–3. The mean SIR of the otosclerosis group had the highest value among the three groups.

**Fig. 3.**
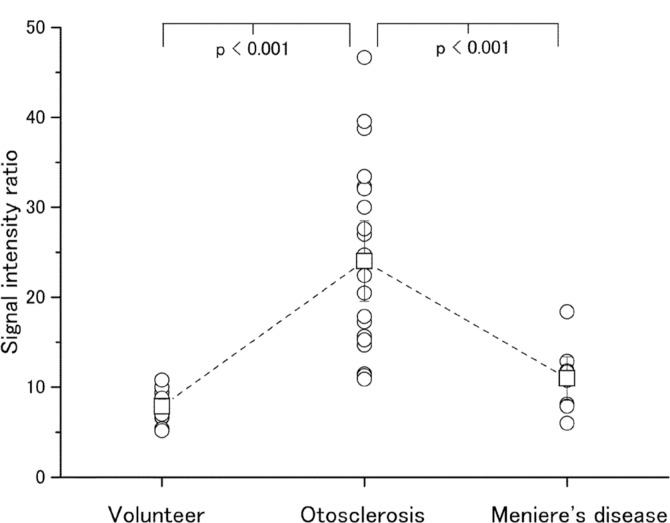
Comparison of the signal intensity ratio (SIR) among the three groups. Open circles show the distribution of the SIR for the volunteer, otosclerosis, and Ménière’s disease groups. The open squares show the means and error bars indicate their 95% confidence intervals. There were statistically significant differences between the volunteer and otosclerosis groups and between the otosclerosis and Ménière’s disease groups in the mean SIR (*P* < 0.001).

Figure 3 shows the distribution of the SIR in the volunteer, otosclerosis, and Ménière’s disease groups. There was a statistically significant difference in the mean SIR among the three groups (*P* < 0.001). Furthermore, based on pairwise comparisons among them, there were statistically significant differences between the volunteer and otosclerosis groups and between the otosclerosis and Ménière’s disease groups in the mean SIR (*P* < 0.001). Although the mean SIR of the Ménière’s disease group was higher than that of the volunteer group, the difference between the volunteer group and Ménière’s disease group did not reach statistical significance (*P* = 0.055).

**Fig. 4.**
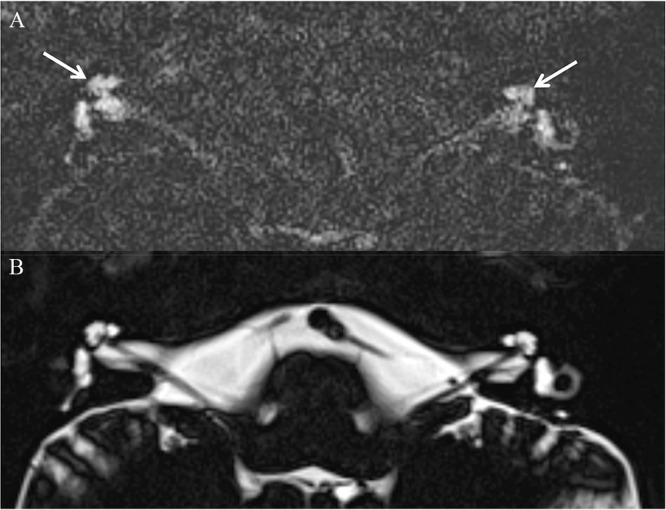
A 31-year-old male volunteer. (**A**) Heavily T_2_-weighted three-dimensional fluid-attenuated inversion recovery (3D-FLAIR) image obtained 4 hours after intravenous administration of a single dose of gadolinium-based contrast agent (IV-SD-GBCA). High signal is seen bilaterally in the cochleae (arrows). In this particular subject, the signal intensity ratio (SIR) was 7.8 in the right cochlea and 7.5 in the left cochlea. (**B**) magnetic resonance cisternography (MRC) at the slice level corresponding to that in (A).

**Fig. 5.**
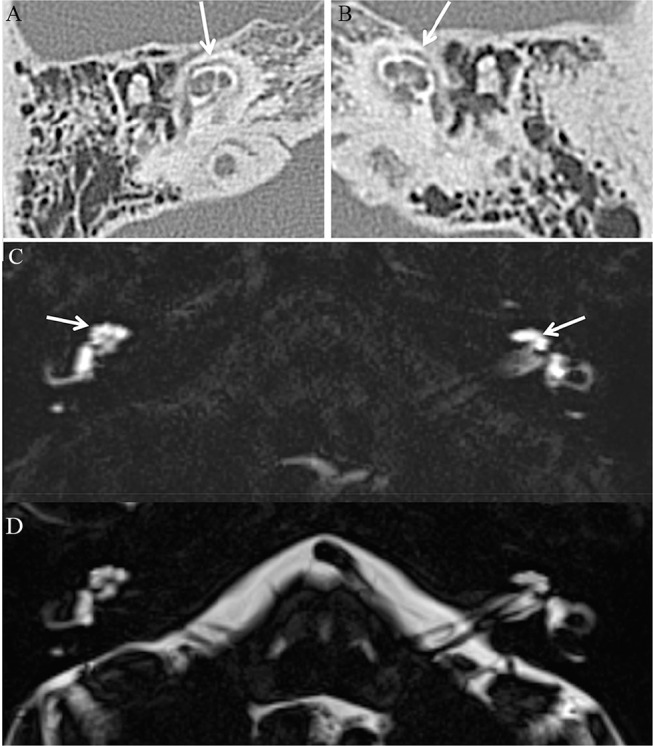
A 53-year-old man with bilateral retrofenestral otosclerosis. (**A**, **B**) On computed tomography images, otospongiotic plaques around the cochleae are visualized as low density bands (arrows). (**C**) Heavily T_2_-weighted three-dimensional fluid-attenuated inversion recovery (3D-FLAIR) image obtained 4 hours after intravenous administration of a single dose of gadolinium-based contrast agent (IV-SD-GBCA). Remarkably high signal is seen bilaterally in the cochleae (arrows). In this particular subject the signal intensity ratio (SIR) was 47 in the right cochlea and 40 in the left cochlea. (**D**) Magnetic resonance cisternography (MRC) at the slice level corresponding to that in (C).

**Fig. 6.**
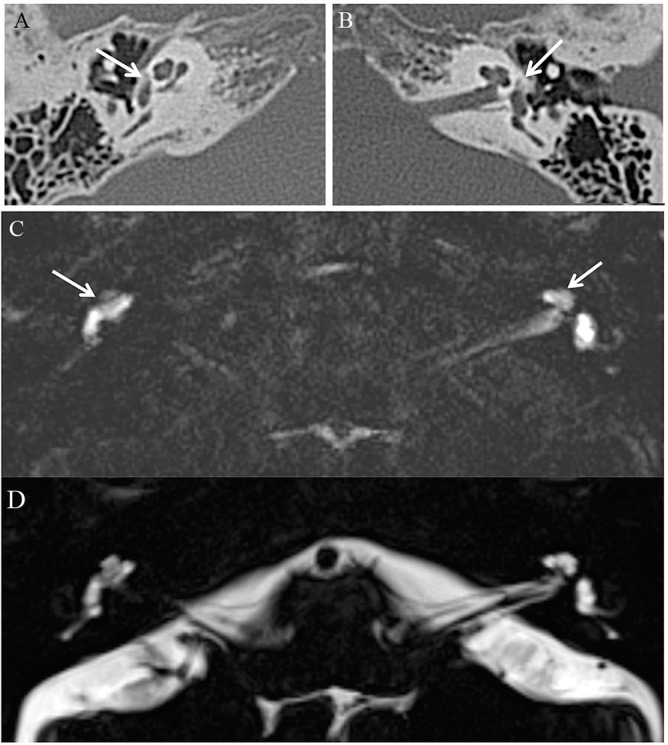
A 27-year-old woman with bilateral fenestral otosclerosis. (**A**, **B**) On computed tomography images, otospongiotic plaques anterior to the oval window are seen as small low density areas (arrows). (**C**) Heavily T_2_-weighted three-dimensional fluid-attenuated inversion recovery (3D-FLAIR) image obtained 4 hours after intravenous administration of a single dose of gadolinium-based contrast agent (IV-SD-GBCA). Remarkably high signal is seen bilaterally in the cochleae (arrows). In this particular subject the signal intensity ratio (SIR) was 27 in the right cochlea and 39 in the left cochlea. (**D**) Magnetic resonance cisternography (MRC) at the slice level corresponding to that in (**C**).

**Fig. 7.**
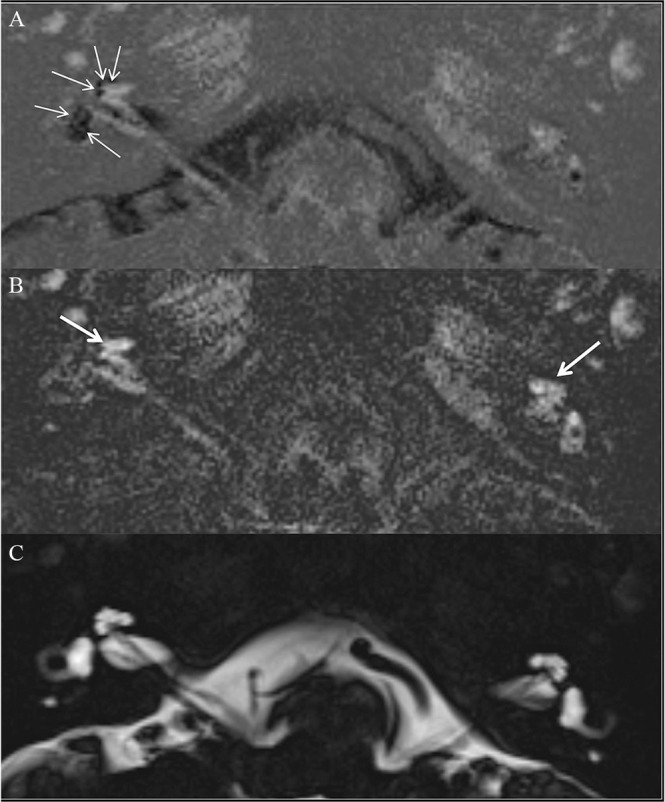
A 28-year-old woman with right Ménière’s disease. (**A**) HYDROPS2 image shows significant endolymphatic hydrops in the cochlea and vestibule (arrows). (**B**) Heavily T_2_-weighted three-dimensional fluid-attenuated inversion recovery (3D-FLAIR) image obtained 4 hours after intravenous administration of a single dose of gadolinium-based contrast agent (IV-SD-GBCA). High signal is seen bilaterally in the cochleae (arrows). In this particular subject the signal intensity ratio (SIR) was 11.7 in the right cochlea and 7.5 in the left cochlea. (**C**) Magnetic resonance cisternography (MRC) at the slice level corresponding to that in (B).

A statistically significant difference was also detected between the fenestral subgroup and retrofenestral subgroup within the otosclerosis group (*P* = 0.033). Representative images are shown in Figs. 4–7.

## Discussion

In the present study, ears with otosclerosis showed a higher SIR on HF images obtained 4 hours after IV-SD-GBCA than ears of the volunteer or Ménière’s disease groups. The retrofenestral subgroup showed a significantly higher SIR than the fenestral subgroup. Retrofenestral otosclerosis is thought to be a more advanced form than the fenestral type.^1,2^ These results suggest that disease progression might be monitored by SIR on HF images obtained 4 hours after IV-SD-GBCM.

In the present study, the mean SIR of the Ménière’s disease group was 39% higher than that of the healthy volunteer group, although the difference in the SIR between the healthy volunteer group and the Ménière’s disease group did not reach statistical significance (*P* = 0.055). In a former study, ears with Ménière’s disease showed 36% higher signal than asymptomatic contralateral ears and the hydrops grade significantly correlated with the contrast effect.^19^ From the results of these studies, it seems as if the SIR in Ménière’s disease is slightly increased compared to the normal state.

Our study has some limitations that should be noted. We did not obtain pre-contrast HF images, thus we cannot rule out the possibility that the high signal observed in HF images obtained 4 hours after IV-SD-GBCA might have existed prior to IV administration. Although we sometimes encounter slightly increased signals in the cochleae of ears with otosclerosis on pre-contrast-enhanced 3D-FLAIR images, the distinct high signal observed in the present study suggests that a large part of the cochlear fluid signal increase is due to increased permeation of GBCA.^4^ Improved detection of signal increase for the cochlear lymph fluid on pre-contrast-enhanced HF images in the patients with sudden sensorineural hearing loss was reported recently.^27^ The cochlear high signal shown on HF images even in the patients with severe sudden hearing loss, is not visually so prominent as that on the images in thepatients with otosclerosis shown in the present contrast-enhanced study. Further study with pre- and post-contrast-enhanced HF images is necessary to firmly determine the degree of contrast enhancement effect in otosclerosis.

We used different GBCM for the volunteers (gadoteridol) and patients (gadodiamide). We cannot completely exclude the possibility that a difference in GBCM might have affected the results of the present study; however, other patient studies using gadodiamide and gadoteridol with the same inversion time for the PEI have been previously reported.^12,28,29^ These studies suggest that the contrast enhancement effects in the perilymph by gadoteridol or gadodiamide are comparable.

We measured the cochlear fluid signal only in the scala tympani of basal turn to avoid the influence of endolymphatic hydrops. Signal measurements in multiple parts in cochlea would be more desirable.

The retrospective nature of this study might have included some selection bias. All otosclerosis patients included in this study were scheduled for stapes surgery. Therefore, we did not include patients in the very early stages of otosclerosis.

In this study, we did not assess the aggressiveness of the otosclerosis by contrast-enhanced T_1_-weighted images and HF obtained immediately after IV-SD-GBCA.^4,5,30,31^ Further study is necessary to correlate the aggressiveness and cochlear fluid signal immediately following and 4 hours after IV-SD-GBCA.

Patients with otosclerosis usually show conductive hearing loss or mixed hearing loss. Occasionally, patients with otosclerosis present with pure sensorineural hearing loss. For patients who present with sensorineural hearing loss and tinnitus, it is possible that HRCT might not be performed and MR imaging for endolymphatic hydrops assessment might be performed instead.^4^ According to the results of the present study, if unusually high cochlear signal intensity is observed on HF images obtained 4 hours after IV-SD-GBCA, otosclerosis might be included on the differential diagnosis list.

Further study is thus warranted to establish the clinical utility of high cochlear signals on HF images obtained 4 hours after IV-SD-GBCA in the diagnosis and monitoring of otosclerosis.

## Conclusion

In patients with otosclerosis, the cochlear SIR on HF images obtained 4 hours after IV-SD-GBCA was significantly higher than in patients with Ménière’s disease or in volunteers. The SIR in the retrofenestral type was higher than that in the fenestral type.

## Figures and Tables

**Table 1.** Summary of volunteers and patients

Healthy volunteer	Age (years)	Gender	Signal intensity ratio	

			Right	Left
1	50	M	7.7	6.7
2	53	M	9.4	5.4
3	31	M	7.8	7.5
4	30	M	10.0	8.1
5	36	M	6.6	5.2
6	38	M	8.1	7.0
7	42	M	8.6	10.8
8	29	M	8.3	8.8
Mean	38.6		8.3	7.4

M, male				

Otosclerosis	Age (years)	Gender	Signal intensity ratio		Type of otosclerosis

			Right	Left	
1	57	F	20.5	op	fenestral
2	44	F	op	15.3	fenestral
3	62	M	24.0	17.3	fenestral
4	62	F	27.0	38.8	fenestral
5	35	M	32.3	32.1	retrofenestral
6	49	F	15.4	17.9	fenestral
7	51	M	30.0	33.4	retrofenestral
8	50	M	24.7	11.5	fenestral
9	53	M	46.7	39.6	retrofenestral
10	47	M	22.4	27.6	retrofenestral
11	39	F	15.6	11.3	fenestral
12	66	M	14.7	10.9	retrofenestral
Mean	51.3		24.8	23.2	

F, female; M, male; op, operated side					

Ménière’s disease	Age (years)	Gender	Signal intensity ratio	

			Right	Left
1	67	F	18.4	na
2	75	M	11.3	na
3	63	M	10.9	na
4	65	M	12.9	na
5	73	F	na	8.1
6	72	M	10.7	na
7	57	F	11.8	7.9
8	24	M	6.0	na
9	38	F	11.7	na

Mean	59.3		11.7	8.0

F, female; M, male; na, non affected side				

**Table 2.** Mean signal intensity ratio (SIR) of the three groups

	Number of ears	Mean SIR	Standard deviation	
Healthy volunteer	16	7.9	1.5	*
Otosclerosis	22	24.0	10.1	*, **
Ménière’s disease	10	11.6	3.4	**

* and **, significant difference (*P* < 0.001)

**Table 3.** Mean signal intensity ratio (SIR) of two subgroups with otosclerosis

	Number of ears	Mean SIR	Standard deviation	
Fenestral type	12	19.9	7.8	*
Retrofenestral type	10	29.0	10.8	*

* , significant difference (*P* < 0.05)
